# Recommendations by a UK expert panel on an aflibercept treat-and-extend pathway for the treatment of neovascular age-related macular degeneration

**DOI:** 10.1038/s41433-019-0747-x

**Published:** 2020-01-03

**Authors:** Adam H. Ross, Louise Downey, Helen Devonport, Richard P. Gale, Ajay Kotagiri, Sajjad Mahmood, Hemal Mehta, Niro Narendran, Praveen J. Patel, Nina Parmar, Nitin Jain

**Affiliations:** 1 University Hospitals Bristol NHS Foundation Trust, Bristol, UK; 2 Hull University Teaching Hospitals NHS Trust, Hull, UK; 3 Bradford Teaching Hospitals NHS Foundation Trust, Bradford, UK; 4 York Teaching Hospital NHS Foundation Trust, York, UK; 5 South Tyneside and Sunderland NHS Foundation Trust, Sunderland, UK; 6 Manchester Royal Eye Hospital, Manchester University NHS Foundation Trust, Manchester, UK; 7 Royal Free London NHS Foundation Trust, London, UK; 8 The Royal Wolverhampton NHS Trust, Wolverhampton, UK; 9 NIHR Biomedical Research Centre at Moorfields Eye Hospital NHS Foundation Trust and UCL Institute of Ophthalmology, London, UK; 10 Bayer plc, Reading, UK

**Keywords:** Retinal diseases, Education

## Abstract

**Objectives:**

This report aims to provide clear recommendations and practical guidance from a panel of UK retinal experts on an aflibercept treat-and-extend (T&E) pathway that can be implemented in clinical practice. These recommendations may help service providers across the NHS intending to implement a T&E approach, with the aim of effectively addressing the capacity and resource issues putting strain on UK neovascular age-related macular degeneration (nAMD) services while promoting patients’ best interests throughout.

**Methods:**

Two structured roundtable meetings of retinal specialists were held in London, UK on 7 December 2018 and 1 March 2019. These meetings were organised and funded by Bayer.

**Results:**

The panel provided recommendations for an aflibercept T&E pathway and developed specific criteria based on visual acuity, retinal morphology and optical coherence tomography imaging to guide reduction, maintenance and extension of injection intervals. They also discussed the extension of treatment intervals by 2- or 4-week adjustments to a maximum treatment interval of 16 weeks, the management of retinal fluid and the stopping of treatment.

**Conclusions:**

The long-term benefits of implementing a T&E pathway may include superior visual outcomes compared with a *pro re nata* (PRN; as needed) protocol, and a lower treatment burden compared with a fixed protocol, which is likely to improve service capacity. Furthermore, the predictable nature of a T&E approach compared with a PRN service may aid capacity planning for the future nAMD treatment demand.

## Introduction

Neovascular age-related macular degeneration (nAMD) is a chronic condition requiring regular monitoring and treatment [1]. The prevalence of nAMD in the UK in 2012 was ~2.4%, with an estimated 39,700 new cases diagnosed per year [2]. Age-related macular degeneration (AMD) remains the leading cause of sight impairment in England and Wales and is therefore associated with a significant disease burden [3].

Increasing age is a risk factor for nAMD, therefore disease prevalence and demand for treatment is expected to rise with an ageing population [1, 2]. The number of nAMD cases in the UK is predicted to rise by 59% from 2015 to 2035 [4]. Growing treatment demand has resulted in increasing strain on the capacity of the AMD service, threatening the delivery of optimal care to patients [1].

Anti-vascular endothelial growth factor (anti-VEGF) treatment is the gold standard of care for patients with nAMD and can be administered using either a reactive or proactive approach [1]. With a reactive, or *pro re nata* (PRN; as needed) approach, patients receive anti-VEGF treatment in response to signs of disease reactivation. However, the success of this treatment paradigm is dependent on regular monitoring and adherence to strict retreatment criteria. In clinical practice, reactive treatment regimens have often led to undertreatment, with patients receiving an insufficient number of injections and, consequently, achieving poorer visual outcomes than seen in clinical trials [5–9]. On a practical level, the unpredictable nature of reactive treatment can hamper capacity planning in AMD services, which can lead to delays in treatment. Treatment delays after diagnosis or disease reactivation can result in clinically significant visual deterioration over time [10]. Furthermore, the need for frequent monitoring visits is burdensome for patients, carers and AMD services [1].

In contrast, proactive treatment protocols involve regular anti-VEGF therapy with the aim of minimising the risk of disease recurrence and/or worsening. The treatment interval can be fixed or individualised to the patient based on morphological changes and visual status. In clinical practice, proactive protocols have been associated with superior visual outcomes to those achieved with a reactive approach [5, 7, 9]. The delivery of intravitreal injections is also more predictable with proactive treatment protocols, which may aid clinic capacity planning [5].

Compared with fixed dosing, the extended treatment intervals of a T&E regimen can lessen the treatment burden for patients and clinics by reducing the number of hospital visits and the overall number of injections [5]. In a T&E regimen, treatment is initiated with a series of monthly loading doses. The treatment interval is then gradually extended until the optimal interval at which to maintain each patient is found. If disease activity recurs, the treatment interval can be reduced depending on specific criteria [5, 11].

Aflibercept is an anti-VEGF therapy approved for use in patients with nAMD. In 2018, it received a product licence extension allowing it to be used in a proactive T&E protocol in Year 1, after the loading phase [12].

Although a T&E regimen, especially when started in the first year, has the potential to relieve capacity issues and optimise resources of often overstretched NHS AMD services, clinicians may not be familiar with the specific requirements for implementation in their service. Additionally, switching a service from a PRN or fixed protocol to a T&E protocol may seem fraught with logistical difficulties.

Currently, the treatment paradigm is shifting from a reactive to a proactive approach. This has created a need for clear recommendations from experts on a T&E pathway that can be implemented in clinical practice. This publication describes recommendations for an aflibercept T&E pathway from two structured roundtable meetings of retinal specialists held in London, UK on 7 December 2018 and 1 March 2019. These meetings were organised and funded by Bayer.

The objectives of this publication are to provide expert-led recommendations for an aflibercept T&E pathway in Year 1 and beyond that can be used in a real-life NHS setting. This practical guidance may help services optimise resources and alleviate capacity issues, thereby ensuring timely treatment for patients and an improved patient experience.

## Expert panel recommendations

### Overview

**Fig. 1:**
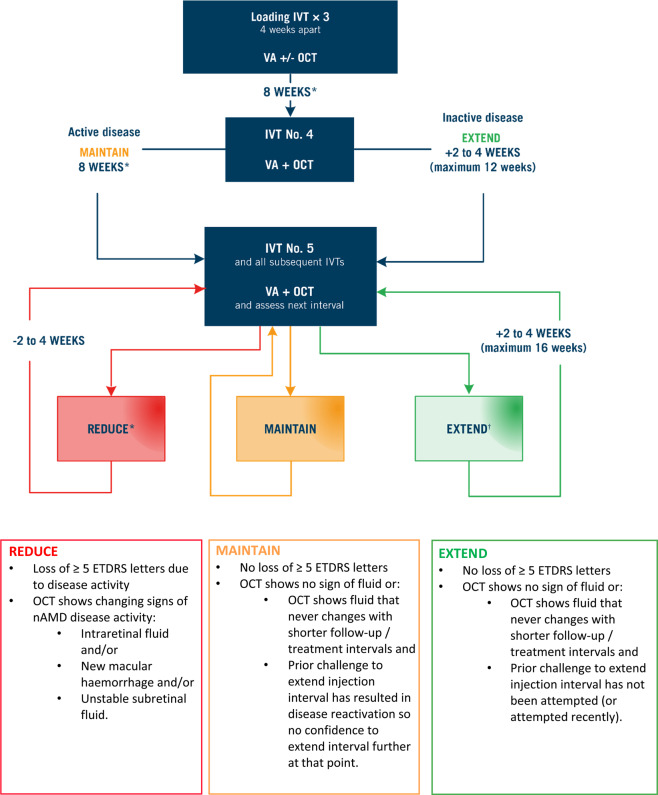
The recommended aflibercept T&E pathway for the treatment of patients with nAMD. Notes: Additional signs of disease activity may include new or worsening subretinal hyperreflective material (SHRM) and changing PED. *Treatment at less than 8-week intervals in the first year is off-label and at the discretion of the treating clinician. ^†^Extension should not be attempted if vision is deteriorating without any other causative pathology in the presence of stable fluid. The decision to extend the treatment interval by 2- or 4-week adjustments is at the discretion of the treating clinician. ETDRS Early Treatment Diabetic Retinopathy Study, IVT intravitreal injection therapy, nAMD neovascular age-related macular degeneration, OCT optical coherence tomography, PED pigment epithelial detachment, T&E treat-and-extend, VA, visual acuity.

The recommended aflibercept T&E pathway is shown in Fig. 1.

The loading phase consists of three consecutive aflibercept doses at 4-weekly intervals. Visual acuity (VA) should be assessed at each visit during the loading phase. The panel suggests using optical coherence tomography (OCT) imaging at Visit 1 to provide baseline data and at Visit 3 to provide early treatment response data; however, the use of OCT during the loading phase is optional and at the treating clinician’s discretion.

Patients receive a fourth aflibercept dose 8 weeks after the third loading dose. Both VA and OCT should be checked at this visit, and at every subsequent visit, to assess the length of the next treatment interval.

According to the licensed posology of aflibercept, three 4-weekly loading injections should be given, followed by a fourth injection 8 weeks later, before treatment interval extension can begin. However, in clinical practice, the treating physician may, at their discretion, choose to maintain or extend treatment intervals immediately after the third injection according to the individual needs of the patient [13].

After the fourth injection, the treatment interval is either maintained with active disease or extended by 2- or 4-week adjustments with inactive disease. After the fifth aflibercept dose, the treatment interval is reduced, maintained or extended based on specific criteria.

### Dosing interval adjustments: extending by 2- or 4-week adjustments

The licensed posology of aflibercept allows adjustment of the treatment interval in either 2- or 4-week increments [13]. Good outcomes with both 2- and 4-week adjustments have been demonstrated in the ALTAIR study, in which patients were randomised 1:1 to receive aflibercept T&E beginning in Year 1, with either 2- or 4-week adjustments to the treatment interval. The maximum treatment interval was 16 weeks. At Week 96, visual outcomes were similar in both the 2-week and 4-week adjustment groups, with mean best-corrected VA (BCVA) gains from baseline of 7.6 and 6.1 Early Treatment Diabetic Retinopathy Study (ETDRS) letters, respectively. Furthermore, 56.9% of patients in the 2-week adjustment group and 60.2% of patients in the 4-week adjustment group achieved an injection interval of 12 weeks or more. The mean number of injections was the same in both groups (10.4 injections over 96 weeks) [14].

As current data do not indicate that either 2- or 4-week increments are superior, the length of these adjustments should be decided by the treating clinician. The expert panel recommends using 2-week increments for most patients to minimise the risk of disease reactivation. However, 4-week adjustments may be considered in patients who respond particularly well and have a dry macula after the loading phase. Occasionally, finer adjustments of the treatment interval may be required, e.g., by 1 week or 3 weeks, at the discretion of the treating clinician.

### Dosing at less than 8-week intervals

Most patients with nAMD tend to have a good response to aflibercept treatment, provided treatment is administered soon after diagnosis and before the onset of end-stage disease, which is characterised by fibrovascular scarring [1, 10]. According to the product licence, the minimum treatment interval with aflibercept in the first year after the loading phase is 8 weeks [13]; however, the panel felt that dosing at less than 8-week intervals may be necessary for some patients in clinical practice. The availability of this option depends on local funding agreements in relation to off-label treatment.

In the Jaffe et al. post hoc analysis of the VIEW studies, 20.3% of patients treated with aflibercept every 8 weeks had early persistent fluid at Week 12, suggesting that more frequent treatment may have been required [15]. Similarly, in the ALTAIR study, treatment interval reductions below 8 weeks were not permitted and at Week 96, 24.8% of participants remained on 8-weekly treatment. This suggests that some patients may have had persistent disease activity throughout the study [14]. Although, in the second year of the VIEW studies, when patients received mandatory dosing at least every 12 weeks, 3.2% of patients originally assigned to the 8-weekly aflibercept treatment arm received between 9 and 11 injections. This suggests that only a small proportion of patients had persistent disease activity requiring treatment more frequently than 8-weekly [16].

In the panellists’ clinical practices, most patients are treated according to the licensed posology for aflibercept, with a minimum treatment interval of 8 weeks after the loading phase in Year 1. The most common reason for more frequent treatment is worsening vision associated with increasing fluid and signs of disease activity on OCT. The decision to treat more frequently than 8-weekly should be based on individual patient need and made on a case-by-case basis after discussion with the patient. The optimal treatment interval changes over time, therefore, dosing at less than 8-week intervals may be a temporary measure, with interval extension possible later on.

### Managing fluid

The expert panel recommends treatment interval reduction with intraretinal fluid (IRF) on OCT, as it is considered a sign of active disease. The experts feel that IRF is generally associated with worse VA outcomes compared with subretinal fluid (SRF). This has been demonstrated by a number of studies, including the CATT (Comparison of AMD Treatments Trials) post hoc analysis, conducted by Jaffe et al., which found that eyes with IRF were associated with worse VA outcomes than eyes without IRF, after both 1 year and 5 years of anti-VEGF treatment [17, 18].

A study by Wickremasinghe et al. demonstrated that eyes with IRF had significantly worse baseline BCVA and, consequently, BCVA outcomes at 12 months than eyes without IRF [19]. Furthermore, a study by Waldstein et al. found that intraretinal cystoid fluid (IRC) was negatively correlated with treatment-naïve BCVA and BCVA outcomes after anti-VEGF treatment [20]. These studies concluded that the presence of IRF at baseline can be considered a biomarker of disease activity, with the potential to predict BCVA in treatment-naïve patients and BCVA outcomes after anti-VEGF treatment [19, 20].

The panellists, however, suggested that IRF may be associated with irreversible retinal structural changes and the loss of normal photoreceptor structure, as seen in cystic degeneration over atrophic retinal fibrosis or outer retinal tubulation. These structural changes may, in some cases, account for the link between IRF and worsening vision, rather than the presence of IRF itself. The experts therefore recommend assessing eyes with persistent IRF for signs of associated retinal structural damage, such as cysts and/or outer retinal tubules, on OCT. Clinicians may be able to tolerate a certain amount of persistent IRF with these signs present, particularly in eyes without macular thickening. A patient with stable IRF for three consecutive visits may be considered for treatment interval extension.

The expert panel recommends treating SRF to stability. The aim should be to achieve dryness; however, in certain circumstances the treatment interval may be maintained or extended with SRF present, provided there are no other signs of disease activity on OCT. Treatment interval maintenance or extension may be appropriate when SRF is persistent and stable despite frequent treatment, or when there is a trend towards improvement over time. The volume of SRF that may be tolerated should be determined by the treating clinician.

**Fig. 2:**
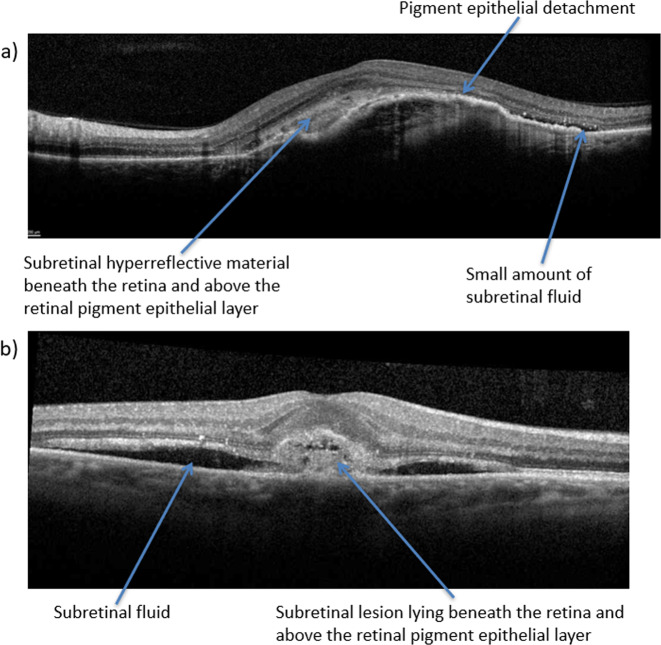
The following OCT images demonstrate different signs of nAMD disease activity, including PED (**a**), SHRM (**a**) and SRF (**b**). nAMD neovascular age-related macular degeneration, OCT optical coherence tomography, PED pigment epithelial detachment, SHRM subretinal hyperreflective material, SRF subretinal fluid.

The treatment interval should always be reduced when persistent SRF is accompanied by new haemorrhage or additional signs of disease activity on OCT, such as new choroidal neovascularisation (CNV) complex, subretinal hyperreflective material (SHRM) or pigment epithelial detachment (PED), as shown in Fig. 2. The treatment interval should also be reduced with new SRF, worsening SRF over time or significant visual loss (≥5 ETDRS letters) that is thought to be due to disease activity and cannot be explained by comorbidities.

These recommendations are evidence-based and supported by various studies. The CATT post hoc analysis found no significant difference in VA at Year 1 between eyes with no SRF, non-foveal SRF or foveal SRF [17]. Similarly, a retrospective analysis of clinical practice in Southampton, in which 255 eyes were treated with aflibercept for one year, found no significant difference in mean BCVA gain between eyes with and without SRF, IRF and/or intraretinal cysts [21].

In the FLUID study, patients treated with a relaxed ranibizumab T&E protocol that tolerated <200 µm SRF achieved VA outcomes comparable to those achieved with an intensive protocol that aimed to resolve all SRF. There was, however, a significant increase in the number of patients extended to and maintained at 12-week intervals at Month 24 in the relaxed group (29.6%) compared with the intensive group (15.0%). There was also a significant reduction in mean injection numbers at Month 24, with 15.8 injections in the relaxed group versus 17 in the intensive group [22]. Although small, this difference would be important for a service with a high treatment burden. Although a direct correlation between SRF and VA has not been found, further evidence is needed to determine the long-term effects of repeated episodes of SRF.

Evidence to date suggests that tolerating some stable SRF does not appear to have a detrimental effect on visual outcomes. This approach could increase the number of patients extended to intervals of >8 weeks compared with a service that does not tolerate any SRF, reducing injection numbers and the treatment burden for patients, while improving AMD service capacity [22].

### Managing disease reactivation

For the purposes of this publication, disease recurrence and reactivation are interchangeable terms used to describe new disease activity in an existing lesion. Signs of new disease activity may include new haemorrhage on fundus examination, new or increasing SRF or IRF, new PED associated with the lesion or new CNV complex not present on previous OCT scans.

A study by Essex et al. in 2016 found that the longer the treatment interval, the higher the risk of disease reactivation for patients managed with an anti-VEGF T&E protocol. The risk of disease reactivation reached 20.9% at 16-week intervals [23]. Although this risk is present, it is managed by the treating clinician and balanced by the benefits of a T&E protocol for both patients and the AMD service; namely, reduced injection numbers, less frequent clinic visits and lower treatment burden compared with a reactive approach.

**Fig. 3:**
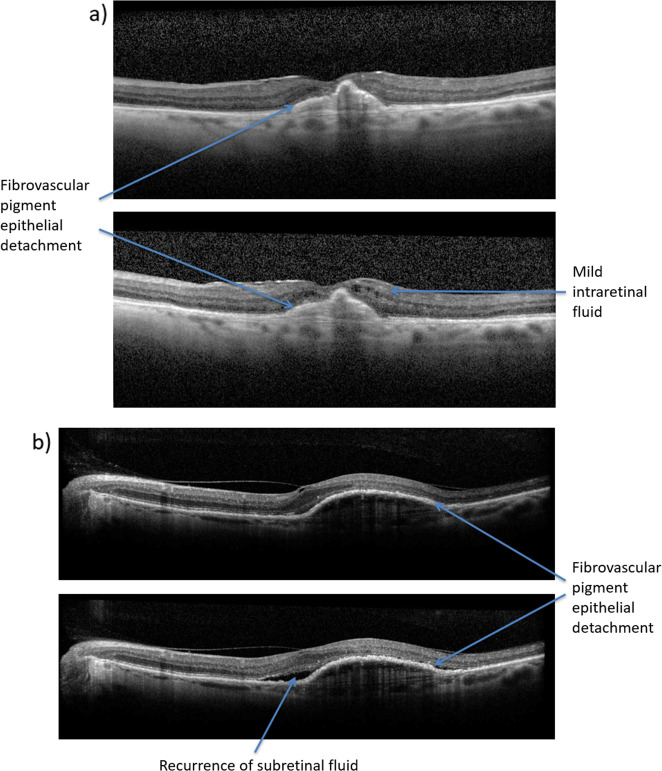
The following OCT images demonstrate cases of minor disease reactivation due to new IRF (**a**) or recurrence of SRF (**b**), which should be treated with a reduction of the treatment interval. IRF intraretinal fluid, OCT optical coherence tomography, SRF subretinal fluid.

The experts recommend treatment interval reduction by 2–4 weeks in patients with mild disease reactivation, as shown in Fig. 3, and reloading in cases of major disease reactivation, at the treating clinician’s discretion. Reloading is defined as three consecutive injections at 4-week intervals and is also used when switching treatments, protocols or when recommencing treatment for new disease activity after stopping treatment.

### Managing fellow eye involvement

nAMD disease activity is usually detected earlier in fellow eyes than first-treated eyes, leading to better baseline VA and maintenance of better VA after prompt intervention [24, 25]. However, any VA loss in the fellow eye has a substantial impact on the patient’s quality of life as they no longer have a non-diseased eye to compensate [25]. Approximately 8–10% of patients with unilateral nAMD develop fellow eye involvement per year [26]. The rate of fellow eye involvement requiring treatment is 42% at 3 years [25].

Disease activity in the fellow eye is often detected while asymptomatic because of regular follow-up of first-treated eyes with OCT monitoring and fundus examination. Therefore, treatment interval extension beyond 8 weeks, as in a T&E regimen, may sacrifice early detection of fellow eye involvement and compromise the VA at presentation. Commencing treatment in fellow eyes with lower baseline VA’s would be expected to result in suboptimal VA outcomes [25, 27].

Patients on >12-week intervals need to be vigilant and should report any signs and symptoms of disease activity in the fellow eye as soon as they become apparent. Both eyes should undergo regular OCT monitoring at each clinic visit to reduce the risk of fellow eye involvement.

Bilateral treatment may be given to manage fellow eye involvement. Often, the eye with more aggressive disease guides the treatment interval, however, this depends on relative visual acuities between the eyes. This may result in overtreatment of the eye with less active disease, but it will reduce the number of clinic visits and the treatment burden for patients compared with attending separate clinic visits for each eye.

With bilateral treatment, each intravitreal injection should be treated as a separate procedure within the same visit, with separate preparation, different equipment and separate vials from two different batches used for each eye. Clinics should therefore always have stock from two batches available [1, 28].

If patients would prefer not to receive bilateral injections, each eye may be treated independently at separate clinic visits. Treatment intervals should be guided by the injection requirements of each eye; however, this will result in more frequent appointments for the patient.

### Stopping treatment

**Fig. 4:**
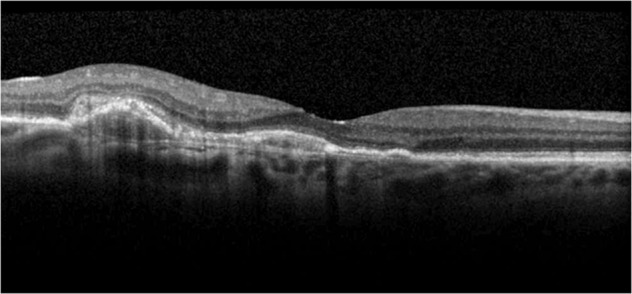
This OCT image shows stable fibrovascular PED with no evidence of disease activity after a period of treatment and successful interval extensions. This patient should be considered for discharge from the AMD service. AMD age-related macular degeneration, OCT optical coherence tomography, PED pigment epithelial detachment.

Patients who are non-responsive to treatment with end-stage disease or fibrosis should be considered for discharge with no follow-up, as shown in Fig. 4. Once discharged, these patients should remain vigilant for VA changes in their fellow eye and a system should be implemented for fast-track re-referral if necessary.

Patients achieving disease stability at the maximum treatment interval, which is considered to be 2–3 consecutive treatments at 16-week intervals, should also be considered for discharge. A two-stage approach should be used to achieve this. First, these patients should be considered for OCT monitoring only. Clinicians should then adopt a monitor-and-extend approach for 12–18 months prior to discharge depending on local arrangements for community support. OCT monitoring may be carried out in the hospital eye service (HES), or after discharge into the community, where patients can be followed up regularly by community optometrists to assess for signs of disease reactivation.

The decision to stop treatment is based on OCT appearance correlating with the presence or absence of functional anatomy. The final decision should be taken upon discussion with the patient. Continued treatment, rather than discharge, may reduce the risk of disease recurrence [29]. The treating clinician may decide that this is the best course of action for patients who have achieved disease stability in their better-seeing eye, reducing the risk of irreversible visual decline.

At present, many discharged patients do not receive regular community OCT monitoring. Regular eye tests and self-monitoring are relied upon for detecting disease reactivation, which are considered by many to be inadequate. Where local service agreements allow, community optometrists may be able to manage long-term OCT monitoring of stable patients who have been discharged; a strategy which is under evaluation in the FENETRE study [30]. This would necessitate ongoing training and medical retina consultant-led quality assurance of community optometrists, a rapid referral mechanism back to the HES, and a system allowing consultant ophthalmologists to access community OCT scans [1].

### Practical guidance for implementing the recommended T&E pathway

Tips for implementing the recommended pathway can be found in Table 1.

**Table 1:** Top tips for implementing the recommended T&E pathway.

• Make sure that all stakeholders, including the business manager, lead clinicians and administrative staff, have an understanding of the benefits of a T&E protocol.
∘ Set up a meeting with the entire team to explain the protocol and answer any questions.
• Make sure there is a designated coordinator and administrative team, distinct from other ophthalmology administrators, to manage appointment bookings, plan for peaks in service and identify any areas of the service that may require additional support.
• Have laminated copies of the locally accepted T&E pathway in all medical retina clinics along with clear discharge guidelines.
• Decide which patients to move to the T&E protocol first, for example treatment-naïve patients.
• Where possible, deliver injections on time to make it easier to identify the optimal treatment interval for each patient.
• If patients miss an appointment, decide whether to extend from the original appointment date or from the rescheduled appointment date.
• Create a robust method for rebooking patients who do not attend their appointment and train staff to use this method.
• Decide how many extensions with disease recurrence should be tolerated, before deciding not to extend to that interval again.
• Plan a policy for managing fellow eye involvement.
• Set up two separate contact numbers, one for booking appointments and one for patients concerned about a sudden deterioration in vision. The latter is especially useful for patients on longer treatment intervals or patients who have been discharged.
• Where possible, visit an established T&E service to see how it is run.

The target time from nAMD diagnosis to treatment is a maximum of 2 weeks in order to preserve visual function [31]. Treatment delays after diagnosis or disease reactivation can result in clinically significant vision loss [10].

The panel makes additional recommendations for services switching from a PRN protocol to the recommended pathway. Treatment-naïve patients, patients requiring frequent injections on a PRN regimen and patients who experience disease recurrence while receiving PRN treatment can be switched to the T&E pathway first. Although patients switching from a PRN regimen will have fewer clinic visits on a T&E protocol, an increase in injection numbers should be anticipated with a proactive approach. Stable patients on long-term follow-up should be monitored in virtual clinics to increase capacity. Temporarily cancelling additional clinic activities when switching protocols may free up team members to administer injections. Alternatively, additional injectors could be recruited to cope with an increased injection demand, particularly in the first year when the treatment burden is higher than in subsequent years [14].

### Closing comments

nAMD prevalence in the UK is increasing, which is expected to continue with the ageing population [2]. This has led to increasing demand for treatment and an increasing strain on AMD services across the UK [1].

The aflibercept pathway detailed here, provides clear recommendations and practical guidance from experts that can be implemented in clinical practice, with the aim of optimising resources and improving the patient experience. T&E protocol benefits may include superior visual outcomes for patients and reduced clinic visits compared with a PRN protocol, and lower overall injection numbers and treatment burden for patients and treating clinicians compared with a fixed protocol [5, 7, 9].

Additional data are required to improve nAMD patient management, including real-world evidence from UK patients treated with an aflibercept T&E protocol in Year 1, data on the proportion of patients requiring less than 8-weekly aflibercept treatment using the disease activity criteria described in this publication, and data on the long-term effects of tolerating SRF and the volume of SRF that can be tolerated safely.

These recommendations provide another nAMD treatment regimen option and may help service providers across the NHS intending to implement a T&E approach. The effort required to implement this pathway in the short term will be outweighed by the long-term benefits seen in clinical practice, which may include improved service capacity. Furthermore, the predictable nature of this pathway, compared with a PRN protocol, is likely to aid capacity planning, helping to meet the increasing nAMD treatment demand in the future.

## Summary

### What was known before

- Growing treatment demand has resulted in increasing strain on AMD (age-related macular degeneration) service capacity, threatening the delivery of optimal care to patients
- The anti-VEGF (anti-vascular endothelial growth factor) treatment paradigm is shifting from a reactive approach to a proactive approach, which may improve patient outcomes, reduce treatment burden and aid clinic capacity planning
- Aflibercept received a product licence extension in 2018 allowing it to be used in a proactive T&E (treat-and-extend) protocol in Year 1, after the loading phase

### What this study adds

- This paper describes clear recommendations from a roundtable of UK retinal experts on an aflibercept T&E pathway that can be implemented in clinical practice
- Specific retreatment criteria developed based on visual acuity, retinal morphology and optical coherence tomography imaging to guide reduction, maintenance and extension of injection intervals
- Practical guidance from experts on how to implement the recommended pathway, with the aim of optimising resources and improving the patient experience
